# Supramolecular assembly of KAT2A with succinyl-CoA for histone succinylation

**DOI:** 10.1038/s41421-018-0048-8

**Published:** 2018-08-07

**Authors:** Yugang Wang, Yusong R. Guo, Dongming Xing, Yizhi Jane Tao, Zhimin Lu

**Affiliations:** 1 Brain Tumor Center, Department of Neuro-Oncology, The University of Texas MD Anderson Cancer Center, Houston, TX 77030 USA; 2 Department of BioSciences, Rice University, Houston, TX 77005 USA; 3 Cancer Institute, The Affiliated Hospital of Qingdao University, Qingdao, Shandong China; 4 Qingdao Cancer Institute, Qingdao, 266061 Shandong China; 5 School of Life Sciences, Tsinghua University, 100084 Beijing, China; 6 Department of Molecular and Cellular Oncology, The University of Texas MD Anderson Cancer Center, Houston, TX 77030 USA; 7 Graduate School of Biomedical Sciences, Houston, TX 77030 USA

Dear Editor,

Histone modifications regulate many fundamental biological processes, including DNA replication, transcription, and repair. Eighteen posttranslational modifications of histones, including acetylation, succinylation, and methylation, have been reported^1–3^. Lysine acetyltransferase 2A (KAT2A, also known as GCN5), a member of the GCN5-related N-acetyltransferase superfamily and a component of Spt-Ada-KAT2A-acetyltransferase (SAGA) and Ada-two-A-containing complexes, was identified as the first transcription-related histone acetyltransferase in 1996^4,5^. KAT2A binds to acetyl-coenzyme A (CoA) and transfers its acetyl group to histones to regulate chromatin architecture and locus-specific transcription^6^.

Our recent studies reported that KAT2A can also function as a histone succinyltransferase by directly transferring the succinyl group from succinyl-CoA to histone H3 lysine 79 (H3K79), which is important for the regulation of gene expression in tumor cells^7^. To elucidate the catalytic mechanism, we determined the structures of both the apo and succinyl-CoA-complexed KAT2A using X-ray crystallography^7^. By comparing these structures with that of KAT2A in complex with acetyl-CoA^7^, we previously demonstrated that succinyl-CoA and acetyl-CoA occupy similar binding sites in the catalytic domain of KAT2A and identified key residues interacting with the acyl chains^7^. In the present work, we report a novel high-order assembly for the catalytic domain of KAT2A observed in the crystal structures of both the apo and succinyl-CoA complexes. It is important to note that both crystals were grown under conditions (pH of 4.6) different from the previously reported crystallization condition (pH of 7.0) of a monomer of the catalytic domain of KAT2A^8^.

Although apo and succinyl-CoA-complexed KAT2A were crystallized in different space groups with different crystal packing contacts (i.e., P4_1_2_1_2 vs. P2_1_3), the same octahedral supramolecular assembly was observed in both crystals: 24 KAT2A molecules formed a spherically shaped complex with a hollow center (Fig. 1a–c). Octahedral assemblies are characterized by 4-fold, 3-fold, and 2-fold symmetry axes. With these symmetry operations, an assembly of 24 molecules is generated from a single protein protomer. In the crystal of apo-KAT2A (i.e., space group P4_1_2_1_2), 24 molecules in each crystallographic asymmetric unit gave rise to a complete octahedral assembly. The crystal of succinyl-CoA-complexed KAT2A had the space group of P2_1_3 with eight molecules in each asymmetric unit, but 24 molecules from three asymmetric units made a complete octahedron of the KAT2A–succinyl CoA complex (Fig. 1a–c).

**Fig. 1:**
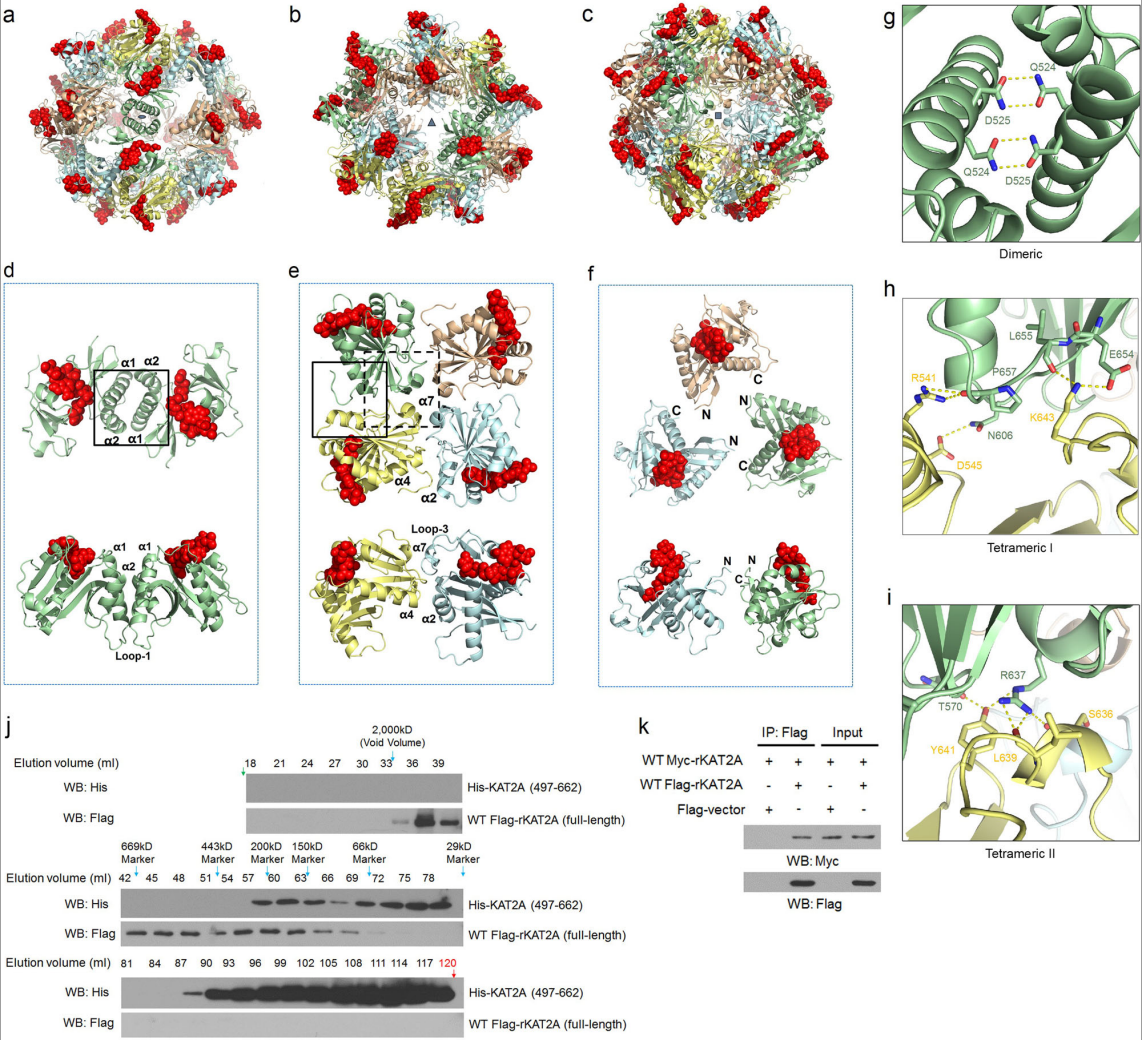
The high-order assembly of KAT2A in complex with succinyl-CoA. **a–c** Octahedral complex of KAT2A in association with succinyl-CoA. The protein molecules are shown as ribbons, whereas the substrate succinyl-CoA molecules are shown in red as space-filling models. The complex is viewed along **a** 2-fold, **b** 3-fold, and **c** 4-fold symmetry axes. **d–f** Three different KAT2A oligomers found in the octahedral assembly. A dimer (**d**), tetramer (**e**), and trimer (**f**) are shown. The upper panels in these three figures represent top views along the symmetry axes from the outside of the octahedron. The lower panels are views from the side, with the molecules at the back removed for clarity. Secondary structure elements at the oligomer interfaces are labeled. **g–i** Hydrogen bonds and salt bridges at the dimeric (**g**) and tetrameric (**h**, **i**) interfaces. The protein molecules are shown as ribbons, whereas the interacting residues are labeled and shown as sticks. The molecular regions shown in **g**, **h**, and **i** correspond to the rectangle box highlighted in **d**, solid rectangle box in **e**, and dashed rectangle box in **e**. **j** Gel-filtration analyses of purified recombinant His-KAT2A (497–662) (upper panel) and wild-type (WT) Flag-rKAT2A (full-length) in the nucleus of 293T cells (lower panel). Bacterially purified His-KAT2A (497–662) or the nuclear lysate from WT Flag-rKAT2A (full-length)-expressing 293T cells was injected into an AKTA Purifier system with a HiPrep 16/60 Sephacryl S-300 High Resolution column for molecular weight-dependent fractionation. The elution from 18 ml to 120 ml was collected with 3 ml for each fraction. Immunoblot analysis of each fraction was performed with the indicated antibodies. The elution volume for each molecular weight marker is indicated. A second peak of His-tagged catalytic domain of KAT2A near 110 ml was observed; this was likely due to the nonspecific interaction of the catalytic domain of KAT2A with the resin. The same peak was not affected by increasing the NaCl concentration from 0.15 M to 0.5 M. **k** WT Myc-rKAT2A was co-transfected with WT Flag-rKAT2A or a control vector into U251 glioblastoma cells. Immunoprecipitation was performed with an anti-Flag antibody. Immunoblot analysis was performed with the indicated antibodies

The stability of a protein–protein interaction usually correlates with the amount of buried surface areas. Based on results from an interface analysis performed by the program PISA^9^, we found that the formation of the KAT2A octahedron structure was mediated by strong interactions at both dimer and tetramer interfaces, with total buried surface areas of 1400 Å^2^ and 1300 Å^2^, respectively (Fig. 1d–f; Supplementary Table S1). At the dimer interface, helices α1 and α2 from one subunit were in close contact with the same two helices from a neighboring subunit (Fig. 1d). The formation of tetramers in the octahedron was largely mediated by α2, α7, and loop 3 of one molecule interacting with α4 and the C-terminal tail of an adjacent molecule (Fig. 1e). Interactions of KAT2A around the three-fold symmetry axis were weak; the buried surface area was less than 10 Å^2^ (Fig. 1f), suggesting that tetramers or dimers may act as precursors for octahedron assembly. Interactions at the dimer interface were mostly hydrophobic in nature, except for two pairs of hydrogen bonds mediated by Q524 and D525 (Fig. 1g). In comparison, more polar interactions were observed at the tetrameric interface, including a salt bridge mediated by E654 and K643, as well as hydrogen bonds mediated by the side chains of R541, D545, R637, and Y641 (Fig. 1h, i).

To determine the physiological relevance of the octahedral structure of KAT2A, we employed gel-filtration analyses and identified high-molecular weight complexes of the purified catalytic domain of KAT2A, ranging from ~29 kD to the fractions that correspond to the molecular weight larger than 200 kD (Fig. 1j). Full-length KAT2A isolated from nuclear fractions of 293T cells gave rise to even larger complexes, with an estimated molecular weight of 94kD, up to a maximal detectable molecular weight (≥2000 kD; Fig. 1j). The wide range of molecular weight distributions may include assembly intermediates expected for an octahedral supramolecular complex. Additionally, immunoprecipitation analysis of cells expressing both full-length Flag-tagged KAT2A and full-length Myc-tagged KAT2A confirmed interaction between individual KAT2A proteins (Fig. 1k), further supporting the notion that KAT2A forms high-order assemblies in cells.

Close inspection of the crystal structure of the catalytic domain of KAT2A indicated that both N- and C-termini are exposed on the outer surface of the octahedron (Supplementary Fig. S1). Therefore, other components of the catalytic complex (i.e., the PCAF-HD and bromodomain of KAT2A) may connect to the catalytic domain of KAT2A and may be situated on the surface of the octahedron without disturbing its overall structural organization. Furthermore, all 24 substrate-binding pockets in the octahedron structure are exposed externally (Fig. 1d–f). Thus, rapid diffusion of either acetyl-CoA or succinyl-CoA in and out of the KAT2A active site is possible during catalysis.

Although yet to be reported for any histone acetyltransferases, supramolecular assemblies of enzymes frequently form and are considered advantageous in capturing substrates^10^. Similarly, the formation of a spherically shaped supramolecular complex with 24 catalytic domains of KAT2A is likely to play such a role. The α-ketoglutarate dehydrogenase (α-KGDH) complex binds to KAT2A in the nucleus and facilitates KAT2A’s succinyltransferase activity by providing local succinyl-CoA, which can overcome the disadvantage of limited succinyl-CoA concentration in the nucleus^7^. With an octahedral assembly of the KAT2A:α-KGDH complex, the substrate-binding site of KAT2A is greatly enriched, thus further facilitating the capture of α-KGDH-produced succinyl-CoA by KAT2A to ensure efficient H3K79 succinylation in vivo. Thus, the supramolecular assemblies of the catalytic domain of KAT2A, high local concentrations of succinyl-CoA generated by the KAT2A-associated α-KGDH complex, and high catalytic activity of KAT2A toward succinyl-CoA^7^ should help compensate for the relatively low nuclear concentration of succinyl-CoA to promote KAT2A-mediated histone succinylation.

Together, our recent publication^7^ and these new structural results for the first time identified a histone succinyltransferase, KAT2A, which was known as a histone acetyltransferase. In addition, we unearthed an important mechanism of histone H3 succinylation, in which KAT2A uses succinyl-CoA locally generated by α-KGDH in the same supramolecular complex to posttranslationally modify histone H3 for gene transcription. Our findings highlight the significance of the metabolic enzyme α-KGDH-coupled KAT2A complex in the regulation of gene expression, tumor cell proliferation, and tumor formation.

## Electronic supplementary material

Supplementary Information

## Electronic supplementary material

**Supplementary Information** accompanies the paper at (10.1038/s41421-018-0048-8).
