# Supplementary material for: Supramolecular assembly of KAT2A with succinyl-CoA for histone succinylation
Source: Cell Discov. 2018 Aug 7;4:47. doi: 10.1038/s41421-018-0048-8 (PMC6079010; doi:10.1038/s41421-018-0048-8)
Supplement: Supplementary file 1 — Supplementary Information [file 41421_2018_48_MOESM1_ESM.pdf]

Materials and Methods

Immunoprecipitation and immunoblot analysis

Proteins were extracted from cultured cells using a modified buffer, and immunoprecipitation and immunoblot analyses with corresponding antibodies were performed as described previously [1]. The band intensity was quantified using the Image Lab software program (Bio-Rad).

Expression and purification of recombinant proteins

Full-length His-KAT2A and the His-KAT2A catalytic domain were expressed in BL21 strain of *Escherichia coli*. The cultures grew at 37°C to an optical density at a wavelength of 600 nm of about 0.6 before treatment with 0.5 mM isopropyl β-d-1-thiogalactopyranoside (IPTG) at 18°C. Cell pellets were collected, resuspended in BugBuster lysis buffer (EMD Millipore, San Diego, CA) with a supplement of cocktail proteinase inhibitors and DNase I, and processed using sonication. Soluble His-tagged proteins were purified using the ÄKTA system with 5 ml of a HisTrap column (GE Healthcare Life Sciences, Pittsburgh, PA). Expression of the His-KAT2A catalytic domain was induced and the domain was purified as described previously [2]. The protein purity was identified via gel staining with GelCode Blue Staining.

Gel Filtration Analysis

One milliliter of nucleus lysate with the concentration of 2mg/ml or the purified His-KAT2A catalytic domain with the concentration of 4mg/ml was injected into an AKTA Purifier system with HiPrep^{TM} 16/60 Sephacryl^{TM} S-300 High Resolution column for molecular weight-dependent fractionation. The nucleus was isolated from the WT Flag-rKAT2A overexpressing HEK293T

cells and lysed with lysis buffer (0.5% Triton X-100, 150mM NaCl, 50mM Tris-HCl, 0.5mM EDTA, pH 7.5, supplemented with protease inhibitor cocktail). The purified his-KAT2A catalytic domain was obtained via the description above.

Crystallization and structure determination

The purified recombinant KAT2A catalytic domain was dialyzed with 20 mM HEPES-NaOH (pH 7.5) and 150 mM NaCl and concentrated to 4 mg/ml for crystallization. For co-crystallization, 10 mM succinyl-CoA was added to the protein solution. Prism-shaped crystals appeared within 1 day in hanging drops containing 1.5 μl of protein solution and 0.5 μl of mother liquor (in 0.1 M sodium acetate, pH 4.6, and 1.4 M ammonium sulfate or 0.5 M lithium chloride and 1.2 M ammonium sulfate). Crystals usually grew to full size in 7 days. KAT2A crystals were frozen in mother liquor substituted with 25% glycerol. X-ray diffraction data on the crystals were collected by the Life Sciences Collaborative Access Team at the Advanced Photon Source (Lemont, IL). Diffraction images were processed using the HKL-2000 software program [3]. For crystal structure determination, the human KAT2A:acetyl-CoA complex structure (Research Collaboratory for Structural Bioinformatics Protein Data Bank ID: 1Z4R) was used for molecule replacement. The acetyl-CoA ligand was removed from the model to reduce model bias. Molecular replacement was calculated using the Phaser software program in the PHENIX software suite [4]. The structure models were then manually adjusted using the Coot software program [5] and refined using PHENIX. Structure figures were prepared using the PyMOL molecular graphics system (version 1.2r3pre). The coordinates were deposited at the Research Collaboratory for Structural Bioinformatics (RCSB) Protein Data Bank.

Supplementary references

1 Lu, Z. *et al.* Activation of protein kinase C triggers its ubiquitination and degradation. *Molecular and cellular biology* **18**, 839-845 (1998).

2 Schuetz, A. *et al.* Crystal structure of a binary complex between human GCN5 histone acetyltransferase domain and acetyl coenzyme A. *Proteins* **68**, 403-407, doi:10.1002/prot.21407 (2007).

3 Otwinowski, Z. & Minor, W. Processing of X-ray diffraction data collected in oscillation mode. *Method Enzymol* **276**, 307-326, doi:Doi 10.1016/S0076-6879(97)76066-X (1997).

4 Adams, P. D. *et al.* PHENIX: a comprehensive Python-based system for macromolecular structure solution. *Acta crystallographica. Section D, Biological crystallography* **66**, 213-221, doi:10.1107/S0907444909052925 (2010).

5 Emsley, P., Lohkamp, B., Scott, W. G. & Cowtan, K. Features and development of Coot. *Acta Crystallogr D* **66**, 486-501, doi:10.1107/S0907444910007493 (2010).

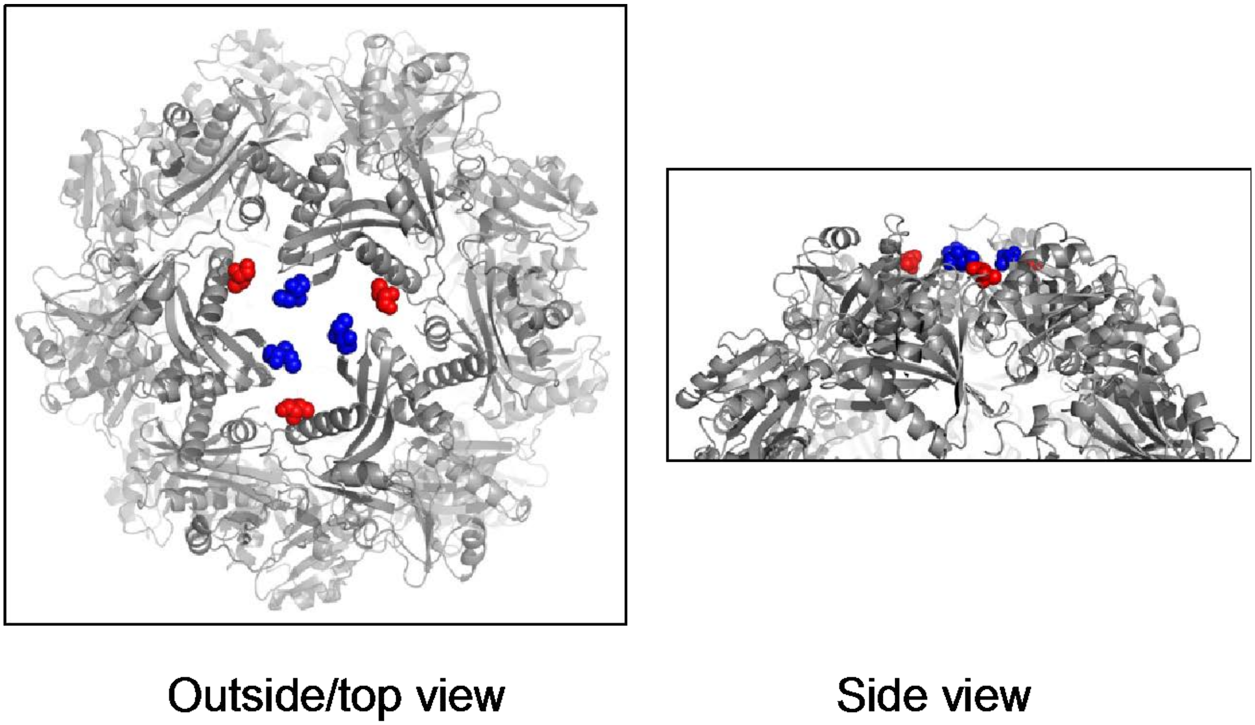

Figure S1. The octahedral complex of the KAT2A catalytic domain with N-/C- termini highlighted. (Left) Top view along the 3-fold symmetry axis. (Right) Side view with the 3-fold symmetry axis in the vertical direction. The residues at N- and C-terminus are shown in blue and red, respectively, as a space-filling model.

Table S1. Octahedral interfaces.

	Dimeric (mol E and mol D)	Trimeric	Tetrameric (mol A and mol B)
**Hydrogen bonds**	E:ASN 525[ND2] D:GLN 524[OE1]<br>E:GLN 524[NE2] D:ASN 525[OD1]<br>E:GLN 524[OE1] D:ASN 525[ND2]<br>E:ASN 525[OD1] D:GLN 524[NE2]	None	A:ASN 606[ND2] B:ASP 545[OD2]<br>A:ARG 637[NH1] B:SER 636[O]<br>A:ARG 637[NH1] B:LEU 639[O]<br>A:ARG 637[NH2] B:LEU 639[O]<br>A:ARG 637[NE] B:TYR 641[OH]<br>A:THR 570[O] B:TYR 641[OH]<br>A:GLU 654[OE1] B:LYS 643[NZ]<br>A:LEU 655[O] B:LYS 643[NZ]<br>A:PRO 657[O] B:ARG 541[NH2]<br>A:PRO 657[O] B:ARG 541[NH1]
**Salt bridges**		None	A:GLU 654[OE1] B:LYS 643[NZ]
